# Therapeutic effect of palbociclib in chondrosarcoma: implication of cyclin-dependent kinase 4 as a potential target

**DOI:** 10.1186/s12964-019-0327-5

**Published:** 2019-02-26

**Authors:** Zhengxiao Ouyang, Sisi Wang, Ming Zeng, Zhihong Li, Qing Zhang, Wanchun Wang, Tang Liu

**Affiliations:** Department of Orthopedics, The Second Xiangya Hospital, Central South University, Changsha, 410011 Hunan China

**Keywords:** Chondrosarocma, CDK4, Palbociclib, Rb, Target therapy

## Abstract

**Background:**

Chondrosarcoma is a malignant cartilaginous neoplasm of the bone which resistant to radiation therapy and chemotherapy. Cyclin-dependent kinase 4 (CKD4) is highly expressed in human cancer, and palbociclib, the inhibitor of CDK4 has been used clinically under FDA approval for application in cancer therapeutic remedies. However, the level of CDK4 and the treatment possibility in chondrosarcoma require further exploration. Thus, we aim to investigate the level of CDK4 and accompanying therapeutic effects of palbociclib in chondrosarcoma.

**Methods:**

We used immunohistochemistric analysis to evaluate human CDK4 productions in chondrosarcoma tissues. The inhibitory expression of CDK4 by siRNA or palbociclib on cell proliferation, invasion, migration, apoptosis and cycle arrest of chondrosarcoma were determined by MTT, wound healing, transwell and flow cytometry. CDK4/Rb signaling pathway were determined by western blot and Immunofluorescence assay. The inhibition effect of palbociclib on tumor growth within the bone were determined by bioluminescence imaging in vivo.

**Results:**

CDK4 was found to express significantly in human chondrosarcoma samples. The enhanced levels of CDK4 were interlinked with malignant metastasis and undesirable prognosis of chondrosarcoma patients. CDK4 was also highly expressed in human chondrosarcoma cell lines and its inhibition by specific siRNA and palbociclib lead to a decrease in cell proliferation, accompanied by the phosphorylation of Rb. Furthermore, palbociclib also induced cell cycle arrest in G1 phase and decreased cell migration and invasion via CDK4/Rb signaling pathway. Administration of palbociclib in vivo could reduce tumor burden in chondrosarcoma.

**Conclusions:**

In summary, these data highlight CDK4 inhibitors, such as palbociclib, as potential promising therapeutics in the treatment of human chondrosarcoma.

## Background

Chondrosarcoma is a malignant cartilaginous tumor, which predominantly affects adults [1], and is the second most commonly seen primary malignant bone tumor, next to osteosarcoma, with an incidence of 1 in 1,000,000 people [2]. Chondrosarcoma presents a wide spectrum of clinical symptoms and can be distinguished from other primary bone tumors by the production of chondroid tissue. Several subtypes with different clinicopathological features are recognized, and about 80–90% of chondrosarcomas are conventional chondrosarcomas, which are notorious for their resistance to conventional chemotherapy and radiation therapy [1, 3]. Thus, the treatment way of a large majority of patients with conventional chondrosarcoma depend solely on surgical resection. If recurrence and metastasis occur, there are no treatment option for patients with inoperable tumors and metastatic lesions. Thus, it is crucial to elucidate the molecular background and the pathways underlying chondrosarcoma to develop new, targeted therapeutic strategies to improve clinical outcome [4–7].

The dysregulation of cell cycle plays crucial roles in cancer development and progression [8]. Cyclin-dependent kinases (CDKs) belong to serine/threonine (Ser/Thr) protein kinases family and regulate the cell cycle through phosphorylation of some substrates. They are frequently found to be highly expressed in malignance [9]. Herein, CDK4 which regulates the G1-S phase cell cycle transition could bind to cyclin D1 (D-type cyclin) for deactivating tumor suppressor retinoblastoma protein (Rb) in cancer cells [10]. Rb, in its phosphorylated form, is shown to activate the expression of transcription factor E2F and allow the initiation of numerous gene productions in modulating cell cycle and cell apoptosis, subsequently leading to cancer progression [11, 12]. The enhanced expression of the cyclin D/CDK4/Rb pathway inevitably contributed to the rampant survival and proliferation of cancer cells within a large variety of malignant categories, especially osteosarcoma [13] and synovial sarcoma [14]. Intriguingly, amplification of 12q13 is a common and consistent genetic aberration in advanced chondrosarcomas and the gene for CDK4 is localized at 12q13 [15]. Thus, CDK4 might be an attractive target for alternative anticancer therapy in chondrosarcoma. Furthermore, the Food and Drug Administration (FDA) recently approved palbociclib (IBRANCE®), a specific CDK4/6 inhibitor, for the treatment of breast cancer [16, 17]. The phase II clinical trial for liposarcoma has already completed and proved to be efficacious with favorable PFS and occasional tumor response [18]. However, the potential role of CDK4 in chondrosarcoma is still not clear and there is no data evaluating palbociclib in chondrosarcoma treatment. Therefore, we determined the expressions of human CDK4 in chondrosarcoma samples and evaluated the treatment effects of palbociclib in a chondrosarcoma xenograft mouse model.

## Main materials and methods

### Human chondrosarcoma cell lines and cell culture

Human chondrosarcoma cell line CS-1 was established in our laboratory as previously reported [19–21] and human chondrosarcoma cell line SW1353 was obtained from the American Type Culture Collection (Rockville, Maryland, USA). Cells were incubated in standard RPMI 1640 medium added with 10% fetal bovine serum (FBS) and 1% penicillin/streptomycin (Hyclone, USA) in a condition of 37 °C, 5% CO_2_.

### Establishment of human chondrosarcoma tissue microarray (hTMA)

This study was carried out in accordance with the recommendations of guiding principles of Human Committee of Central South University and approved by the Human Committee of Central South University. All subjects gave written informed consent in accordance with the Declaration of Helsinki. The formalin-fixed, paraffin-embedded tumor specimens of 79 chondrosarcoma tissues from 79 patients were selected from the Second Xiangya Hospital of Central South University. We accumulated a diameter of 0.5 mm core biopsies of individual tissue block on the basis of results from relevant hematoxylin and eosin (HE) histologic analyses. Patients included were enduring chondrosarcoma diagnostically and under therapeutic strategies in the range from 1995 to 2015 at the Second Xiangya Hospital of Central South University. Patients data were accumulated clinically such as age, gender, tumor location, pathological grade, relapsed, metastatic steps, the observation duration plus patients’ prognoses.

### Assessment of human chondrosarcoma tissue slides

The levels of CDK4 were assessed via immunohistochemical analyses following instructive protocols. Specifically, the sectioned samples were heated at 60 °C prior to the xylene-based deparaffinization. Subsequently, slides were moved through various concentrations of ethanol graded. Following the retrieval of surface epitope and the suppression of endogenous peroxidase, the slides were blocked. The primary antibody of human CDK4 (Cell Signaling Technology, Beverly, MA, USA) was used at 4 °C overnight. Next, relevant binding antibodies, DAB reagents and hematoxylin QS was employed to indicate the expressions of CDK4 and the nuclei of chondrosarcoma cells. All the samples were kept under VectaMount AQ for protection and visualized with Olympus microscope (Olympus, PA, USA).

Three separate pathologists were employed to evaluate and score the results of collected samples. They were remained unknown about the features of tumor and patient information regarding samples. By determining the number of cancer cells of evident nucleus labeling, the levels of CDK4 were classified as six categories: negative nuclear labeling was level 0; positive labeling less than 10% was level 1; positive labeling between 10 and 25% was level 2; positive labeling between 26 and 50% was level 3; positive labeling between 51 to 75% was level 4; positive labeling more than 75% was level 5. We identify cancers scoring at least 3 as high CDK4 level and less than 3 as low CDK4 level.

### Extraction of protein and Western blotting

We used radioimmuno-precipitation assay (RIPA) lysis buffer added with phenylmethysulfonyl fluoride (PMSF) to extract proteins (Beyotime biotechnology, China). A BCA Protein Assay Kit (Thermo Scientific, IL, USA) was deployed to quantify the amount of extracted protein. Western blot was then conducted based on previous report [22]. Specifically, 30 μg of lysates were separated with SDS-PAGE precast gel and then transferred to an activated polyvinylidene fluoride (PVDF) membrane. After the blockage by 5% non-fat milk powder, primary antibodies against human CDK4, phospho-Rb, human Rb and β-Actin (Cell Signaling Technology, MA, USA) were administered to membranes respectively at 4 °C overnight. Next, the membranes were rinsed with Tris-buffer saline containing 0.05% Tween (TBST) for several times, prior to the incubation of secondary antibodies of IRDye 800CW or IRDye 680LT at room temperature. Further, the membranes were washed again, and Odyssey Infrared Imaging System (Li-COR Biosciences, NE, USA) was deployed to visualize the bands. The Odyssey Software 3.0 was used to measure the quantification of bands.

### Immunofluorescent observation

Chondrosarcoma CS-1 and SW1353 cells seeded in 6-well plates were fixed with 4% paraformaldehyde (PFA), permeabilized with methanol and blocked by 1% bovine serum albumin (BSA). Subsequently, tumor cells were administered with relevant CDK4 or β-Actin primary antibody (Cell Signaling Technology, MA, USA), prior to the administrations of corresponding secondary immunofluorescent antibody (Alexa Fluor 488[green]/594[red]) (Invitrogen, NY, USA). Next, immunofluorescently-stained tumor cells were visualized with fluorescence microscope (Nikon, Japan).

### The transfection of siRNA and palbociclib administration

We used both specific CDK4-targeted siRNA and palbociclib administration to down-regulate the expression of CDK4 in chondrosarcoma cells. The siRNA sequence of CDK4 used was 5′-CUCUUAUCUACAUAAGGAU-3′. With respect to the transfection of siRNA against CDK4, CS-1 and SW1353 cells were both transfected with escalated dosages of presynthesized CDK4 siRNA (0, 20, 40, 60 nM) plus Lipofectamine 2000 transfection reagent (Invitrogen, CA, USA). Herein, the negative control of siRNA at 60 nM was utilized. With respect to palbociclib administration, CS-1 and SW1353 cells were treated with various dosages of palbociclib (0, 0.1, 0.3, 1.0, 3.0 μM) for up to 6 days, prior to the following exploration.

### Determination of cell viability

Upon the transfection of CDK4 siRNA for 3 days, as well as palbociclib administration for 2, 4, 6 days, MTT experiment was deployed to investigate the cell viability of tumor cells. After corresponding treatments, cells were administered with MTT solution, followed by the further incubation at 37 °C for 3 h. Subsequently, the mixed solution of formazan with 100 μl acid isopropanol was added into wells. The optical density (OD) at a wavelength of 490 nm was determined.

### Assessment of cell metastatic ability

We assessed the cell migration and invasion rates to determine cell metastatic ability. Wound healing assay was used to investigate the capability of cell migration. The cell layer of confluent tumor cells that seeded in 6-well plates (5 × 10^5^ cells/well) was scraped by two straight lines parallelly via a pipette tip. Subsequently, cells were further treated with palbociclib (1 μM) for starvation of 72 h with culturing medium containing 2% FBS. The image of the wounds after treatments of palbociclib for 0, 16, and 32 h was visualized to measure the distance between two edges of scraped wound at five locations independently of each picture. Following formula was deployed then to calculate the ability of cell migration: (scratch width at the beginning - scratch width in the end) / 2.

With respect to the evaluation of invasive capability, transwell assay using a culturing chamber precoated with Matrigel (100 g/ml, 100 μl/well) was employed as previously described [23]. Briefly, the top chambers were seeded with CS-1 or SW1353 cells at 3 × 10^3^ cells/well with 1 μM palbociclib while the lower chambers were added with 500 μl complete medium containing 10% FBS. Upon further stimulation of 12 h, 4% PFA and 1% crystal violet was used to fix and stain the penetrated invasive tumor cells respectively. Finally, an inverted microscope was used to obtain positive images, and Image-Pro Plus 6.0 was utilized to calculate the invasive tumor cells.

### Evaluation of cell fate

Cell fate of cell cycle and apoptosis in CS-1 and SW1353 cells was measured by flow cytometry. With respect to the evaluation of cell cycle, we harvested tumor cells for fixation with 70% ethanol, prior to the administration of RNase A (Thermo Scientific, NY, USA) for 0.5 h. Propidium Iodide (Sigma-Aldrich, MO, USA) was used to stain cells for 0.5 h. Flow cytometry analyses were then deployed to investigate the cell fate and MultiCycle software (Phoenix Flow Systems, CA, USA) was employed to count the cellular events in each cell cycle stage. With respect to the exploration of cell apoptosis, we harvested tumor cells for further staining with FITC annexin V and Propidium Iodide (Invitrogen, NY, USA) for 0.5 h. Flow cytometry was used to analyze the effect of palbociclib on cell apoptosis.

### Treatment with palbociclib in vivo

This study was carried out in accordance with the recommendations of guiding principles of Animal Care Committee of Central South University and protocol was also approved by the Animal Care Committee of Central South University. The chondrosarcoma cells SW1353 were transduced with a lentiviral luciferase expression vector as previously reported [24]. After clonal selection, luciferase-expressing clones were selected for inoculation into an animal model. Briefly, SW1353 cells were transduced with the lentiviral supernatant (kindly provided by Dr. Eric Kaijzel from LUMC) with dextran of 1 mg/ml for 4 h. Following transduction, we screened tumor cells with puromycin of 2 mg/ml for luciferase activity. Cultured human chondrosarcoma cell SW1353-LUC was adjusted to 1 × 10^7^ cells/ml in sterile PBS. A total of 15 mice (BALB/c nu/nu, 6 weeks old) were inoculated with SW1353-LUC cells directly into the tibiae plateaus of the mice percutaneously. All mice were classified as three groups randomly (*n* = 5 for each group): vehicle group (0.9% saline), palbociclib groups (75 or 150 mg/kg/day). Palbociclib was administered intraperitoneally for 28 days, and then the mice were sacrificed. A Xenogen IVIS 200 imaging system (Caliper Life Science) was deployed to investigate the malignant progress of transplanted tumors by bioluminescence imaging for up to 4 weeks. Bone lesions were evaluated by BLI intensity. Expression of CDK4 was evaluated by immunohistochemistry. Body weight was recorded once a week to evaluate cachexia.

### Data analyses

We used SPSS 13.0 software (Statistical Package for Social Science, Chicago, IL, USA) to perform the statistical analyses. All of the data obtained were presented in Means ± S.D. An analysis of variance (ANOVA) was used to evaluate the statistical significance of obtained results. Kaplan-Meier method and the log-rank test were deployed for survival analysis and significance assessment respectively. We considered a *P* value ≤0.05 as statistically significant.

## Results

### The expression of CDK4 was associated with prognosis of chondrosarcoma clinicopathologically

**Fig. 1:**
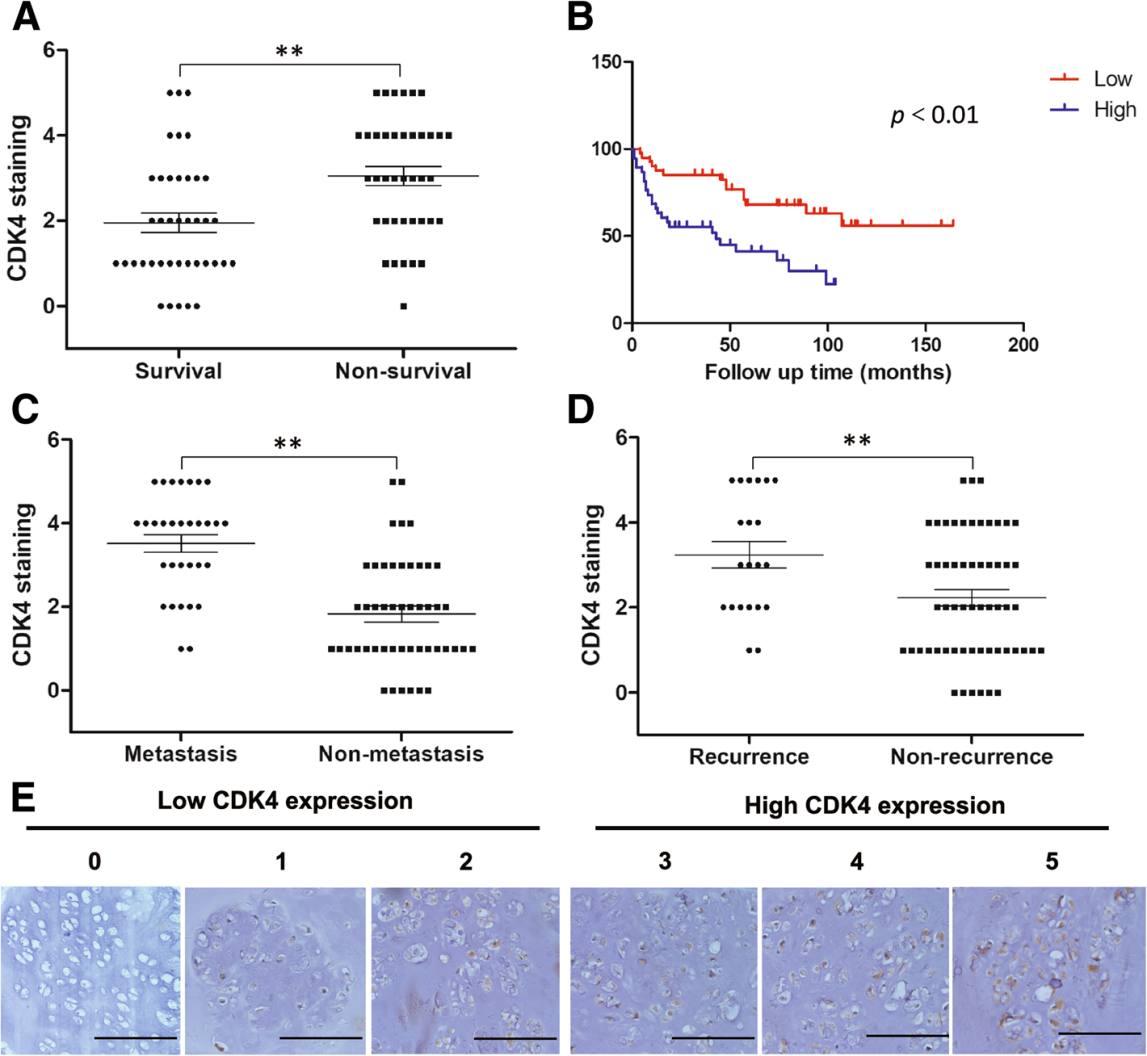
CDK4 expression levels are associated with the clinicopathological characteristics of chondrosarcoma patients. (**a**) Distribution of CDK4 staining scores in the chondrosarcoma tissue samples from surviving and non-surviving patients. (**b**) Kaplan-Meier survival curve of sarcoma patients with high staining (≥ 3) or low staining (< 3) for CDK4. Distribution of CDK4 staining scores in the chondrosarcoma tissue samples from patients with and without metastasis (**c**), patients with and without recurrence (**d**). ** means *P* < 0.01 compared with the former group. (**e**) Representative images of different immunohistochemical staining intensities of CDK4 (Original magnification 200X, scale bar =100 μm). On the basis of the percentage of cells with positive nuclear staining, CDK4 staining patterns were categorized into 6 groups: 0, no nuclear staining; 1+, < 10% positive cells; 2+, 10–25% positive cells; 3+, 26–50% positive cells; 4+, 51–75% positive cells; 5+, > 75% positive cells. Tumors with a staining score of ≥3 were designated as high CDK4 expression and < 3 were designated as low CDK4 expression

To explore the vital roles of CDK4 in chondrosarcoma, we determined the expression of CDK4 in human chondrosarcoma tissues. The vivid crosstalk that related the expression of CDK4 to the malignant features plus treatment effects of chondrosarcoma patients, was also evaluated. CDK4 productions were classified on the basis of the scoring system. The scores ≥3 were regarded as high production levels. As shown in Fig. 1, CDK4 was shown in the nucleus of chondrosarcoma cells. From the 79 samples analyzed, the expressions of CDK4 were found in 73 (92.4%) cases positively. During the follow-up observation of up to 162 months, the expressions of CDK4 in survivor tissues were remarkably lower than those from non-survivors (Fig. 1A). The results of Kaplan–Meier survival analysis demonstrated the more desirable prognosis for CDK4 low-staining patient than CDK4 high-staining patient (Fig. 1B). More importantly, CDK4 expression levels were also associated with the metastasis and recurrence stage of chondrosarcoma. In Fig. 1C and D, the staining of CDK4 in chondrosarcoma tissues from metastasis and relapsed patients were markedly stronger than that from patients without metastasis and recurrence, respectively. Nonetheless, barely connection was shown to interlink CDK4 expression with patient age, gender, tumor location, tumor volume or pathological grades (Table 1).

**Table 1:** The clinical parameters of chondrosarcoma tissue microarray

Parameters	Number
Gender	
Male	41
Female	38
Age, years	
<45	23
45-60	25
>60	31
Grade	
1	18
2	30
3	6
Dedifferentiation	25
Tumor Location	
Extremities	43
Pelvis	19
Other Locations	17
Tumor Volume	
≤100	36
>100	43
Metastasis Status	
Metastasis	31
Non-metastasis	48
Local Recurrence	
Recurrence	21
Non-recurrence	58
Prognosis	
Survival	40
Non-survival	39

### Down-regulation of human CDK4 inhibited chondrosarcoma cell growth

**Fig. 2:**
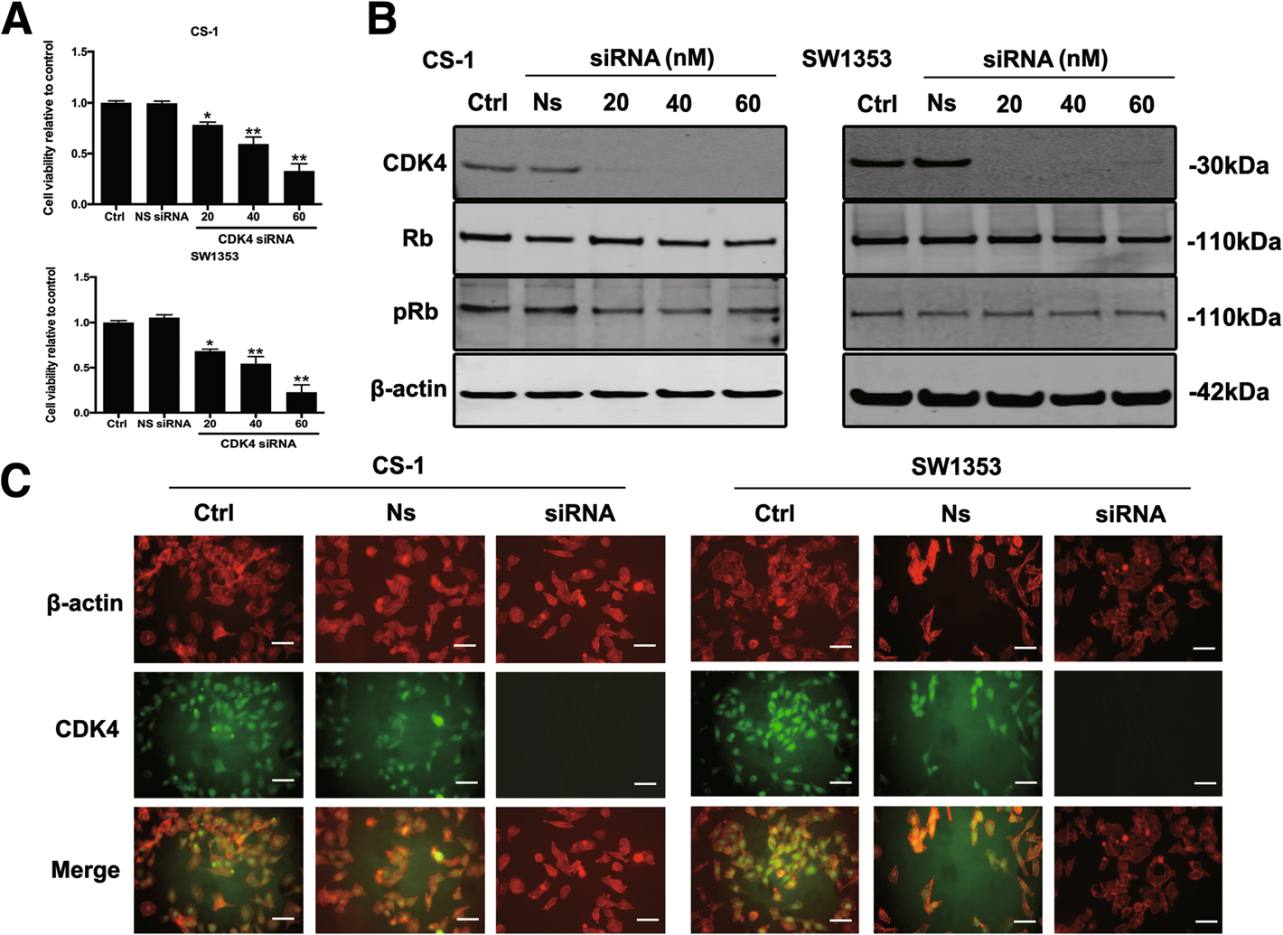
CDK4 is highly expressed in human chondrosarcoma cell lines and CDK4 silencing decreases cell proliferation and suppresses the phosphorylation of Rb. (**a**) Human chondrosarcoma cell lines CS-1 and SW1353 were transfected with increasing concentrations of CDK4 specific siRNA or nonspecific siRNA for 3 days, and cell viability was determined by MTT assay after siRNA transfection. *P < 0.05, **P < 0.01 compared with control group. (**b**) The expression of respective proteins in CDK4/Rb-apoptosis signaling pathway was examined by western blotting. (**c**) Human chondrosarcoma cell lines CS-1 and SW1353 were transfected with 60 nM CDK4 specific siRNA or nonspecific siRNA or Control for 3 days, and expression of CDK4 in CS-1 and SW1353 was assessed by immunofluorescence with antibodies for CDK4 and Actin (scale bar =50 μm). Cells were visualized under a fluorescence microscope after incubation with secondary fluorescent conjugated antibodies Alexa Fluor 488 goat anti-rabbit IgG (green) or Alexa Fluor 594 goat anti-mouse IgG (red)

Due to the close relationship between CDK4 expression and the prognosis of patients with chondrosarcoma, we then explored its expression in human chondrosarcoma cell lines and the potential roles of CDK4 in human chondrosarcoma cell growth. Firstly, we used specific CDK4 siRNA to down-regulate chondrosarcoma CDK4 level for cell viability evaluation. In Fig. 2A, the inhibition of cell viability was dose-dependent, increasing with the addition of escalating CDK4 siRNA dosages in both CS-1 and SW1353 cells, and such inhibitory effect was not observed in cells transfected with nonspecific siRNA. Thus, we next examined the CDK4/Rb signaling pathway in both cells. The western blot showed that CDK4 was highly expressed in both cell lines and siRNA knock-down of CDK4 markedly attenuated the level of CDK4. Following CDK4 silencing, there was a decrease of pRb expression in both cell lines, while the upstream Rb expression failed to be affected (Fig. 2B). Furthermore, for confirmation of the production of CDK4 as well as investigate its position in chondrosarcoma cells subcellularly, we employed immunofluorescence analysis in CS-1 and SW1353 cells. Herein, in Fig. 2C, CDK4 expression molecule was largely limited to the nucleus for both chondrosarcoma cell lines and was suppressed by siRNA treatment in both cell lines.

### Attenuation of CDK4 by palbociclib inhibited human chondrosarcoma cell growth in an Rb-dependent manner

**Fig. 3:**
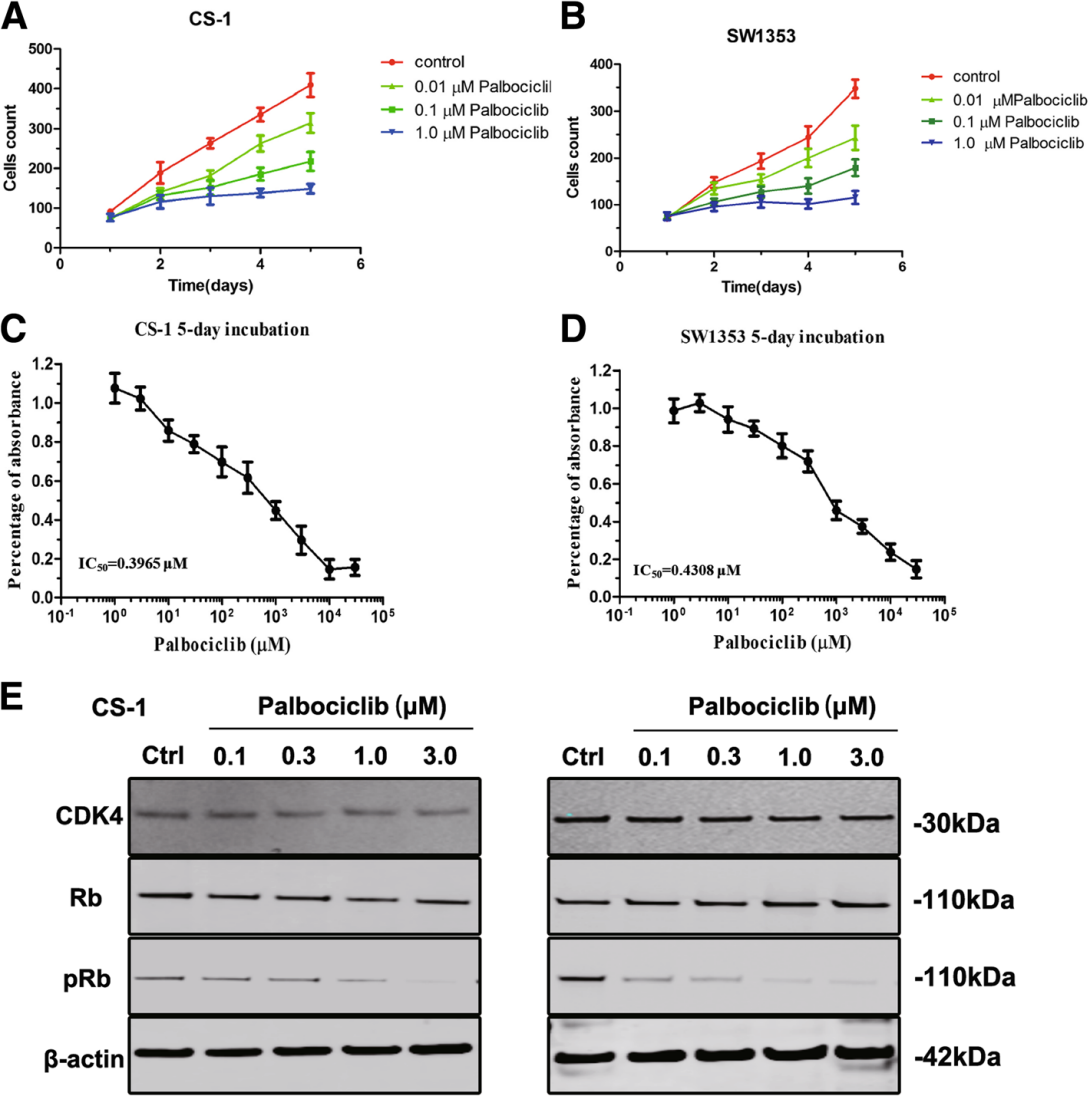
CDK4 inhibition induced by palbociclib decreases cell proliferation in an Rb-dependent manner. (**a** and **b**) Human chondrosarcoma cells CS-1 and SW1353 were exposed to increasing concentrations of palbociclib for 5 days, cell viability was measured by MTT assay. (**c** and **d**) Human chondrosarcoma cells CS-1 (**a**) and SW1353 (**b**) were treated with increasing concentrations of palbociclib for 5 days, and cell growth was analyzed by cell counting. (**e**) Human chondrosarcoma cell lines CS-1 and SW1353 were incubated with increasing concentration of CDK4 inhibitor, palbociclib, for 48 h, and the expression of respective proteins in CDK4-Rb-apoptosis signaling pathway was examined by western blotting

Upon the validation of clinical significance of CDK4 in both chondrosarcoma samples and cell lines, we further assessed CDK4 selective inhibitor, palbociclib, in the treatment of chondrosarcoma. As shown in Fig. 3A and B, the cell viability of both CS-1 and SW1353 cells were decreased in a dose-dependent manner after exposure to increasing concentrations of palbociclib for 5 days. We further determined the IC50 values for palbociclib in both cells after treatment of escalating dosages of palbociclib for 5 days. Results showed the significant decrease of cell viability dose-dependently in CS-1 and SW1353 cells, as exemplified by the values of IC50 of 0.3965 and 0.4308 μM in CS-1 and SW1353 cells, respectively (Fig. 3C and D). In order to further explore the expression of CDK4/Rb signaling after downregulated CDK4 under palbociclib treatment, we screened several associated proteins after the treatment of palbociclib. In Fig. 3E, results showed that after three days of palbociclib intervention, the expressions of pRb were down-regulated significantly, whereas the total Rb expression was not significantly altered. To be noted, palbociclib has been proved to affect the activation of CDK4/6 instead of generation [13], thus in our results, it had no influence on the expression of CDK4.

### Inhibition of CDK4 by palbociclib impeded the progress of chondrosarcoma cell fate

**Fig. 4:**
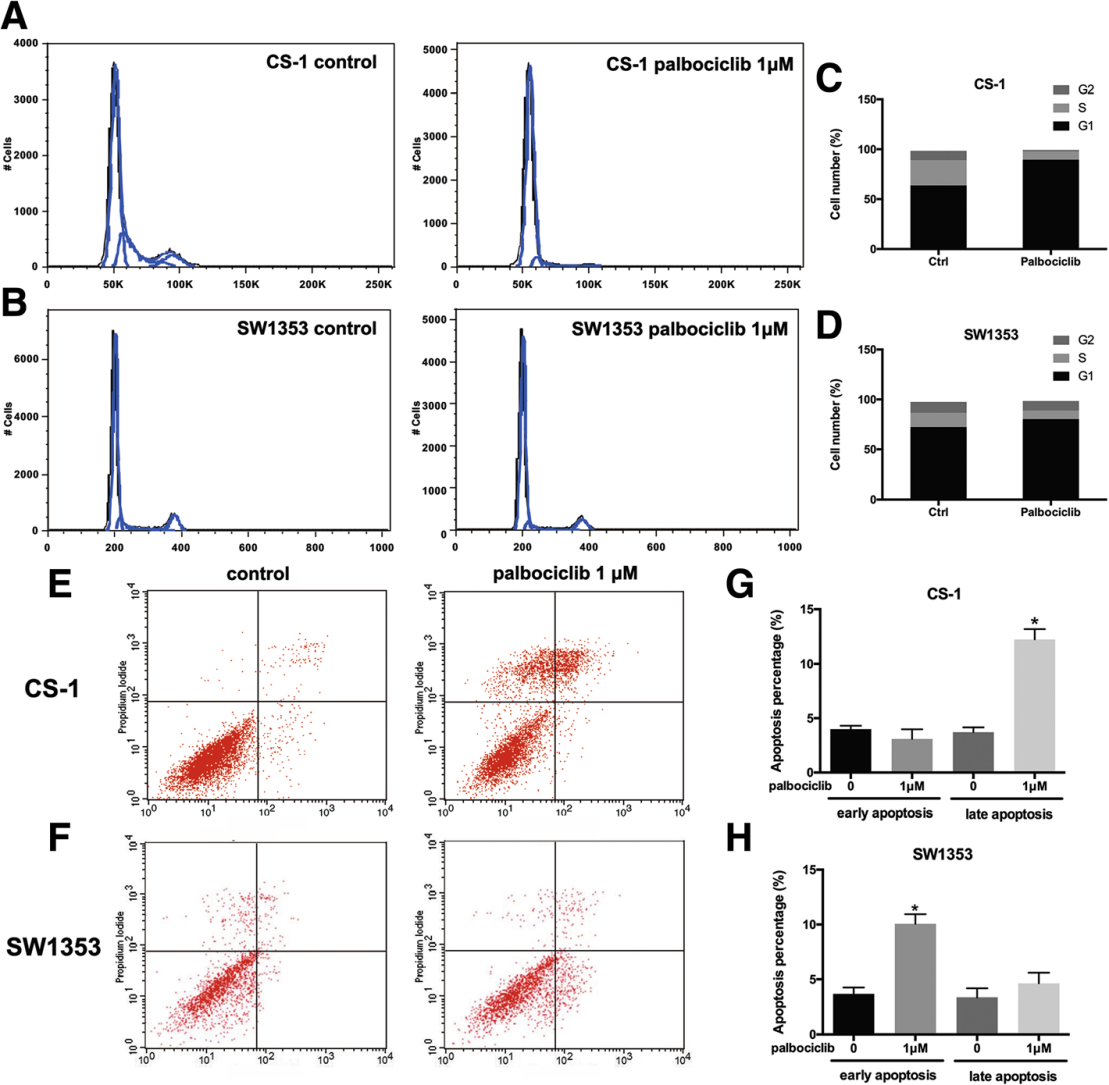
CDK4 inhibition by palbociclib induced cell cycle arrest and apoptosis in chondrosarcoma cell lines. Human chondrosarcoma cells CS-1 and SW1353 were treated with increasing concentrations of palbociclib for the indicated time. After exposure to palbociclib (1 μM) for 24 h, the cell cycle was assessed by flow cytometry analysis. Representative images of cell cycle distribution in CS-1 (**a**) and SW1353 (**b**) with or without palbociclib treatment. (**c** and **d**) Different cell cycle phase rates were analyzed. (**e**) Flow cytometric analysis of palbociclib-treated CS-1 and SW1353 cells. (**f**) Percentage of apoptotic CS-1 and SW1353 cells

It was shown that CDK4/Rb signaling pathway plays an important role in the cell cycle [12]. In order to evaluate the potential regulatory molecular action of mechanisms that inhibiting chondrosarcoma cell survival and proliferation after CDK4 targeting treatment, the effect of palbociclib on progress of the cell cycle and apoptosis was examined. Because of the pronounced cell proliferation inhibition with palbociclib at 1 μM, we employed the same dose of palbociclib for cell cycle and apoptosis examination. Flow cytometry analysis demonstrated an obvious G1-arrest, as evidenced by the decrease of cell events in S phase in CS-1 (Fig. 4A and C) and SW1353 cells after palbociclib treatment for 24 h (both *P* < 0.01) (Fig. 4B and D), indicating the attenuation of CDK4 was capable of facilitating the cell cycle arrest of chondrosarcoma cell in G1 stage, thereby abrogating DNA synthesis. Furthermore, cell apoptosis evaluation showed that, after 48 h treatment with palbociclib, a significantly increased apoptosis rate can be observed in both CS-1 and SW1353 cells compared with the control group (both *P* < 0.01) (Fig. 4E to 4H). Late apoptosis could be observed in the CS-1 cell line, and early apoptosis was observed in the SW1353 cell line. These results demonstrate that inhibition of chondrosarcoma cell proliferation by CDK4 suppression is associated with cell cycle arrest and apoptosis.

### Knock-down of CDK4 inhibited the metastatic ability of human chondrosarcoma cells in vitro

**Fig. 5:**
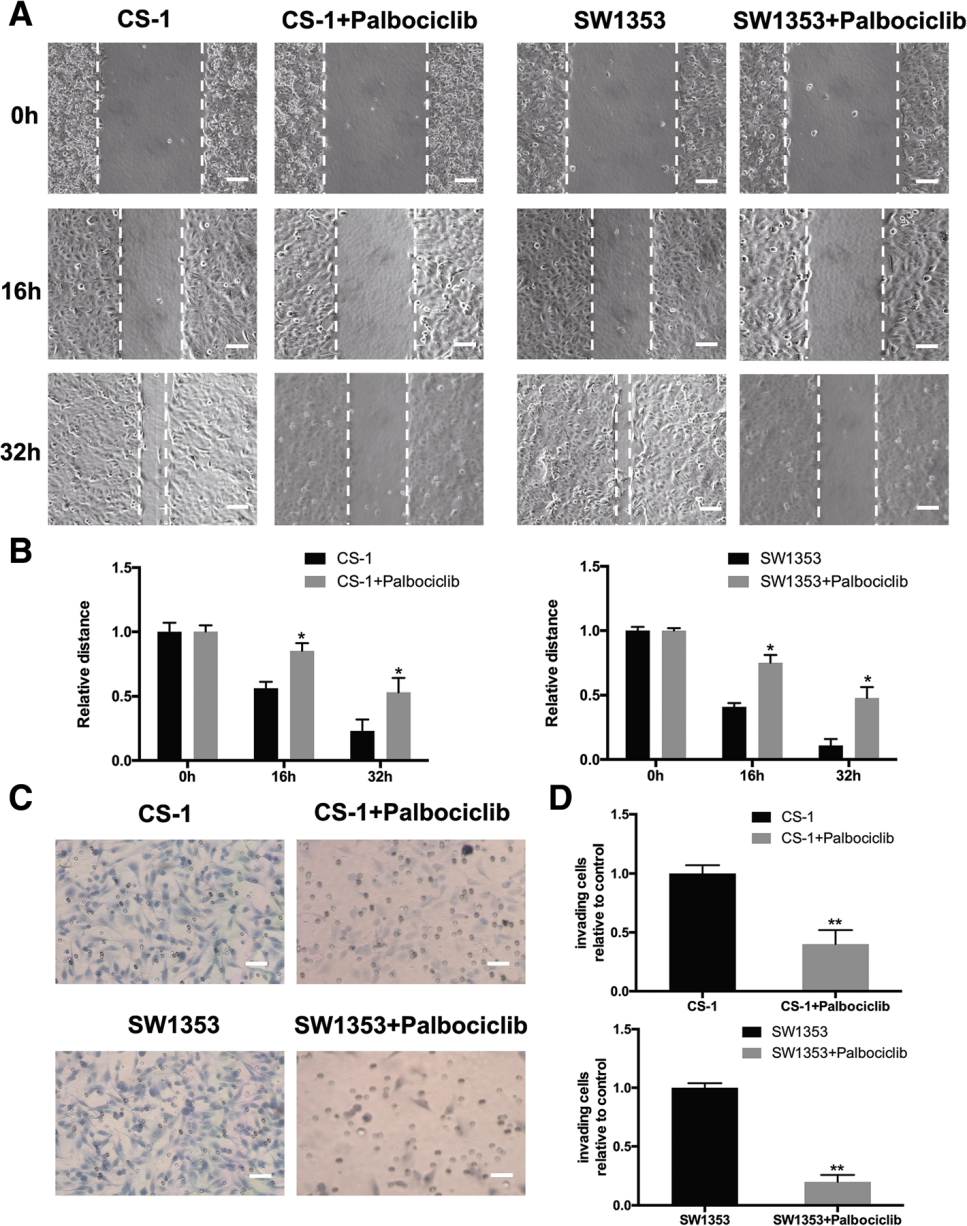
CDK4 inhibition induced by palbociclib suppresses cell migration and invasion. After exposure to 1 μM of palbociclib for the indicated time, the cell migration of CS-1 and SW1353 cells was determined by wound healing assay. (**a**) Representative images of CS-1 and SW1353 cell migration after palbociclib treatment for 0, 16, and 32 h (scale bar =50 μm). (**b**) Cell migration distance of CS-1 cells was measured after palbociclib treatment. *P < 0.05 compared with 0 h group. (**c**) Human chondrosarcoma cells CS-1 and SW1353 were starved for 12 h, and then seeded in the top chambers of transwells with matrigel in the presence of the indicated doses of palbociclib. The bottom chambers of the transwells were filled with a medium containing 10% FBS. Cancer cells were allowed to invade for 10–12 h. The invading purple-stained cells showing irregular shape were photographed and counted (scale bar =50 μm). (D) Quantitative analysis of the percentage of cell invasion using ImageJ. Columns represent the means of experiments performed in triplicate, where the bars represent the SD. **P < 0.01 compared with cell only group

The ability of tumor cells to migrate and invade is the most important factor that contributed to the advanced metastasis of cancer [23]. Since the results from TMA assay indicated that the level of CDK4 was dramatically correlated with the metastasis stage of patients suffered with chondrosarcoma, we therefore investigated the vital significance of CDK4 in the migration and invasion of chondrosarcoma cells in vitro. The migration ability of both cell lines was tested by the wound-healing assay. After treatment with 1 μM of palbociclib, as shown in Fig. 5A, the migration distance of cancer cells were suppressed time-dependently in CS-1 and SW1353 cells in 16 h and 32 h groups. A quantitative test of the relative distance migrated showed that, in comparison with the non-palbociclib treated cells, the migrated distance in the palbociclib treated group was much longer (Fig. 5B, *P* < 0.01). The effect of palbociclib on cell invasion was tested by transwell assay. After exposure to 1 μM of palbociclib for 12 h, the number of invading purple-stained cells was less than that in groups without palbociclib in both cell lines (Fig. 5C). Collectively, these results indicate that the migration and invasion activities of human chondrosarcoma cells were inhibited by palbociclib.

### CDK4 inhibition by palbociclib reduces tumor burden in vivo

**Fig. 6:**
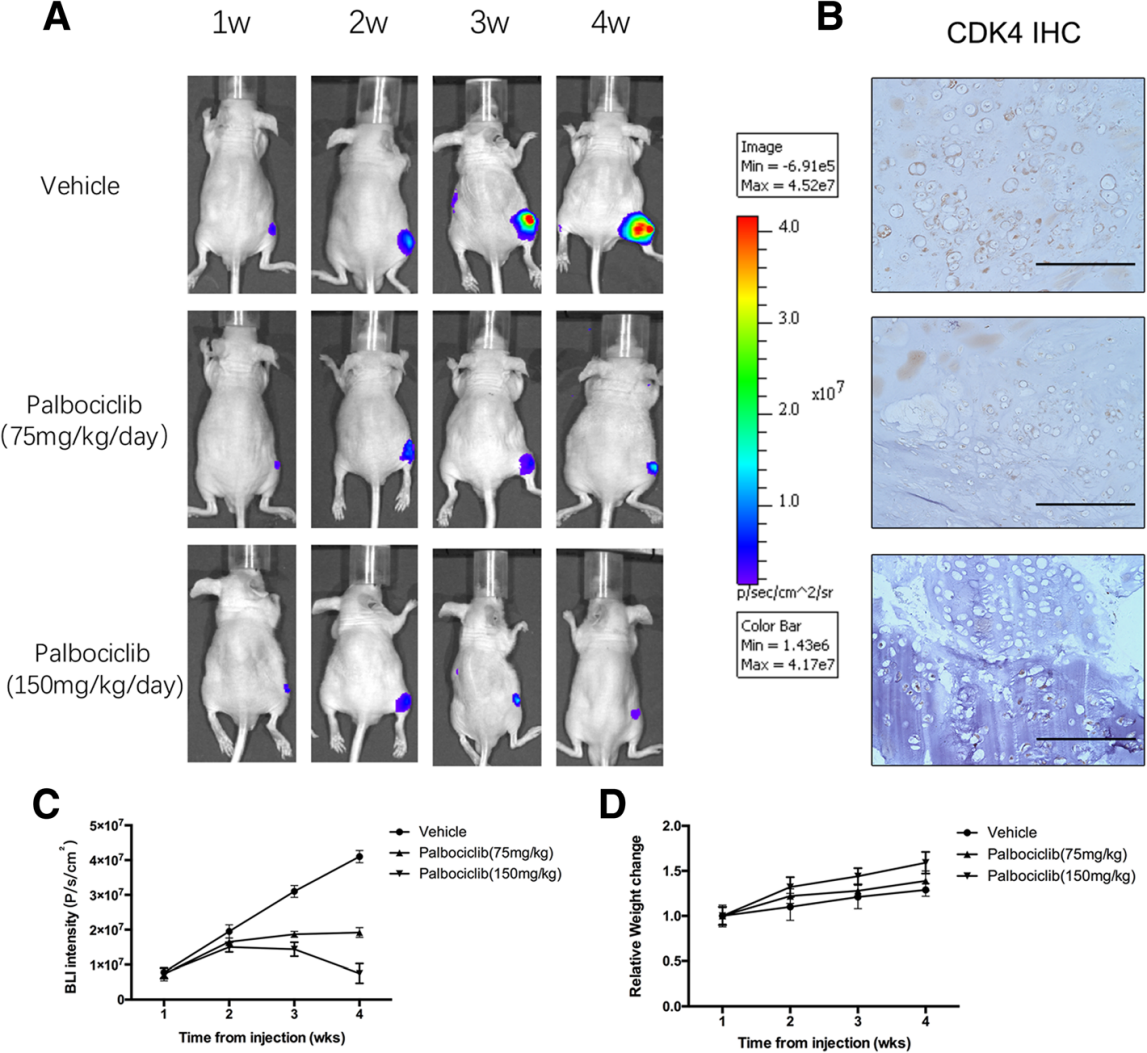
Inhibition of the CDK4 by palbociclib reduces tumor burden in vivo. Six-week-old female BALB/c nu/nu mice were intratibially injected with SW1353 stably transfected with luciferase in PBS. From day 1 post-cell inoculation, animals were treated with the vehicle (PBS) or palbociclib (75 mg/kg/day or 150 mg/kg/day) until day 30. (**a**) Representative live images and (**b**) CDK4 IHC staining of decalcified bones of the mice treated with vehicle (above) or palbociclib (below) at day 30. (**c**) BLI signals were analyzed and quantified using Living Image 3.0 from week 1 to 4 post cell inoculation. (**d**) Evaluation of cachexia (relative body weight)

In light of our findings on the clinical chondrosarcoma samples and the pre-clinical inhibitory effects of CDK4 inhibition by palbociclib against tumor proliferation, migration and invasion, we hypothesized that palbociclib might suppress tumor development in vivo. To test this hypothesis, we constructed an animal model by intratibia injection of SW1353-Luc chondrosarcoma cells, as previously described [24]. As shown in Fig. 6A, all mice injected with chondrosarcoma cells developed bone lesions in the hind limbs at 1 week after cell injection, as determined by bioluminescence. In the vehicle-treated group, the BLI signals of tumors were increased gradually during 4 weeks observation, implicating the progressive advance of malignant tumor growth. However, the intensity of BLI signals of palbociclib-treated group (75 mg/kg/day) failed to increase suggesting growth arrest. In the high concentration palbociclib (150 mg/kg/day) group, tumor progress was observed in the first two weeks after injection. However, after 3 weeks of palbociclib treatment, the tumor burden reduced indicating an anti-tumor effect. The CDK4 IHC results also indicated that CDK4 expression was suppressed with tumor reduction (Fig. 6B). Mean BLI staining values for mice on palbociclib were quantified and the results were consiSstent with our in vitro finding (Fig. 6C). The weight change results show that with tumor reduction, the weight of mice was increased in palbociclib treated group. Thus, our results indicated that palbociclib could reduce chondrosarcoma tumor burden.

## Discussion

In bone and soft tissue sarcomas, such as osteosarcoma [13], liposarcoma [25], rhabdomyosarcoma [26] and chordoma [22], amplification of CDK4 has been associated with poor prognoses. In chondrosarcoma, previous studies reported that expression of CDK4 was associated with high-grade chondrosarcoma in both patients’ samples and cell lines, which suggests an important role in the pathogenesis and treatment of chondrosarcoma [27, 28]. Consistent with these findings, in our study, CDK4 expression was observed in most of the tested tissue samples. Further study on the relationship between the clinicopathological characteristics of chondrosarcoma and CDK4 expression indicated the potent correlation between CDK4 expressions and the therapeutic prognosis of chondrosarcoma patients clinically.

Previous studies on Rhabdomyosarcoma show that CDK4 is essential for sarcoma cell survival and growth, in which knockdown of CDK4 lead to abrogate proliferation of Rhabdomyosarcoma [26]. In chondrosarcoma, previous study showed the shRNA-mediated down-regulation of CDK4 expressions led to a dramatic attenuation in terms of cell viability and growth among OUMS27, SW1353 and CH2879 cell lines [27]. Consistent with these finding, we also showed the positive staining of CDK4 in all investigated chondrosarcoma cell lines (CS-1 and SW1353), indicating the nucleus location of CDK4 by immunofluorescence staining. By specifically knocking down CDK4 expression using siRNA, chondrosarcoma cell growth and phosphorylation of Rb were both suppressed. We also noticed that CDK4 siRNA transfection could abrogate the phosphorylated levels of downstream Rb molecule in CS-1 and SW1353 cells significantly.

Targeting the ATP binding site of CDK4, Palbociclib was the first clinical approved anti-tumor CDK4 inhibitor [12]. Recently, it was investigated as targeted treatment for bone and soft tissue sarcoma, such as osteosarcoma [13], chordoma [22], synovial sarcoma [29] for its availability for (pre)clinical use. In our study, addition of palbociclib in the culture system lead to inhibition of cell proliferation with reduced expression of pRb. Since the treatment of palbociclib normally contributed to the inhibition of CDK4 activity instead of expression level, we used western blot analysis to show that palbociclib failed to affect the level of CDK4 while reducing pRb expression significantly. Collectively, we illustrated that attenuation of CDK4 expression could suppress chondrosarcoma cell proliferation in a CDK4/Rb dependent manner.

Previously, research on CDK4 reported that CDK4 exerts its functional role mainly through the potentiated transition from G1 to S stage during the cell cycle progress [30, 31]. Thus, we examined cell cycle and apoptosis to explore the possible regulative mechanism of chondrosarcoma cell proliferation arrest after CDK4 abrogation. Results obtained indicated that chondrosarcoma cells were inhibited in G1 stage following CDK4 abrogation and CDK4 inhibition contributed to the significant cell apoptosis effectively. Therefore, it is manifested that CDK4 inhibition by palbociclib treatment alleviates the survival and proliferation of chondrosarcoma cells via the enhanced promotion of cell apoptosis by G1 cell cycle arrest. Palbociclib might be a promising candidate for chondrosarcoma treatment.

Up to 71% of patients with high-grade chondrosarcoma develop metastatic lesions of which the 10-year overall survival decreases lower than 29% [32, 33]. In TMA analysis, a high level of CDK4 relates to higher clinical stages and poorer patient prognosis, which indicates the necessity of CDK4 in tumor metastasis. As cell migration and invasion are pivotal steps in cancer cell metastasis and we observed the inhibitory effect of palbociclib on CDK4/Rb signaling in chondrosarcoma. Further, the effect of palbociclib-mediated inhibition of CDK4 expression on the migration and invasion activity of chondrosarcoma was assessed. Surprisingly, palbociclib in non-lethal concentration could inhibit both cell migration and invasion, which led us to hypothesize that this curative effect might also play a role in vivo. There are many kinds of chondrosarcoma mouse models at present, such as the subcutaneous or orthotopic models [34, 35]. As human chondrosarcoma typically occurs in the medulla of the bone, we chose a mouse model with intratibial injection of the tumor cells, which mimicked the human pathology to the best extent. An orthotopic chondrosarcoma mouse model based on the direct injection of luciferase transduced SW1353 chondrosarcoma cells in the bone has been developed, which can be used to closely monitor tumor growth within the bone by bioluminescence imaging. In this animal model, we found that palbociclib could decrease the tumor burden in the bone as demonstrated by lower BLI intensities of mice treated with palbociclib compared with control group. This inhibitory effect of palbociclib might be due to the inhibition of proliferation, invasion, and migration of chondrosarcoma cells. Moreover, injection of palbociclib resulted in a significant decrease in cachexia, which further proved palbociclib might be a promising candidate agent for chondrosarcoma treatment.

There were some limitations in our study. For genomic alterations, the amplification of 12q13 and deletion of 9q21 are two consistent genetic aberrations [15]. Only the CDK4/Rb pathway was tested in our study, others which localized at this region, such as negatively regulating p53, mouse double minute 2 (MDM2), CDK6, cyclin dependent kinase inhibitor 2A (CDKN2A), coding for the tumor suppressor proteins p16, were not examined, as well as natural inhibitors of CDK4, p21 and p27. This is because some studies reported that these mutations are only found in a subset of mainly high-grade central chondrosarcomas. Only the CDK4/Rb upregulation is present in nearly all high-grade central chondrosarcomas and might have the greatest potential for targeted treatment [28]. There are also many new targets for precision therapy, such as Bcl-2 family members [36], which could increase chemosensitivity of the tumor, and survival protein kinases such as the HIF1a [37], Src [38] and PI3K [39] pathways. Further study is needed on these new inhibitors.

## Conclusions

Collectively, we show the significant positive-expression of CDK4 in chondrosarcoma. Also, the enhanced expression of CDK4 is intimately correlated with malignant metastasis and unpleasant prognosis of chondrosarcoma patients. The attenuation of CDK4 inhibits chondrosarcoma cell viability via downregulation of the CDK4/Rb signaling pathway. Palbociclib could also induce cell cycle arrest and apoptosis, as well as suppress migration and invasion of chondrosarcoma cells. Such inhibitory effect could also be observed in vivo. Our results indicate the significant role of CDK4 as potential therapeutic target during chondrosarcoma treatment. Additionally, palbociclib could serve as a novel and promising candidate remedy for the future treatment against chondrosarcoma clinically.

## Abbreviations

ANOVA: An analysis of variance
BLI: Bioluminescence imaging
CKD4: Cyclin-dependent kinase 4
FBS: Fetal bovine serum
FDA: Food and Drug Administration
HE: Hematoxylin and eosin
MTT: 3-(4,5-dimethyl-2-thiazolyl)-2,5-diphenyl-2-H-tetrazolium bromide
PMSF: Phenylmethysulfonyl fluoride
PVDF: Polyvinylidene fluoride
Rb: Retinoblastoma protein
TBST: Tris-buffer saline containing 0.05% Tween
TMA: Tissue microarray
