# Correction: therapeutic effect of palbociclib in chondrosarcoma: implication of cyclin-dependent kinase 4 as a potential target

**DOI:** 10.1186/s12964-023-01046-y

**Published:** 2023-02-20

**Authors:** Zhengxiao Ouyang, Sisi Wang, Ming Zeng, Zhihong Li, Qing Zhang, Wanchun Wang, Tang Liu

**Affiliations:** Department of Orthopedics, The Second Xiangya Hospital, Central South University, Changsha, 410011 Hunan China

**Correction: Cell Communication and Signaling (2019) 17:17** 10.1186/s12964-019-0327-5

Following publication of the original article [1], the authors identified that Fig. 5C image was uploaded in error where the original data did not match the uploaded data. We suspect that this error was due to the plate handling. Although the results and conclusion are still the same, we would like to correct this error. The corrected Fig. 5C is presented in this correction article. The authors declare that these corrections do not change the results or conclusions of this paper.

**Fig. 5:**
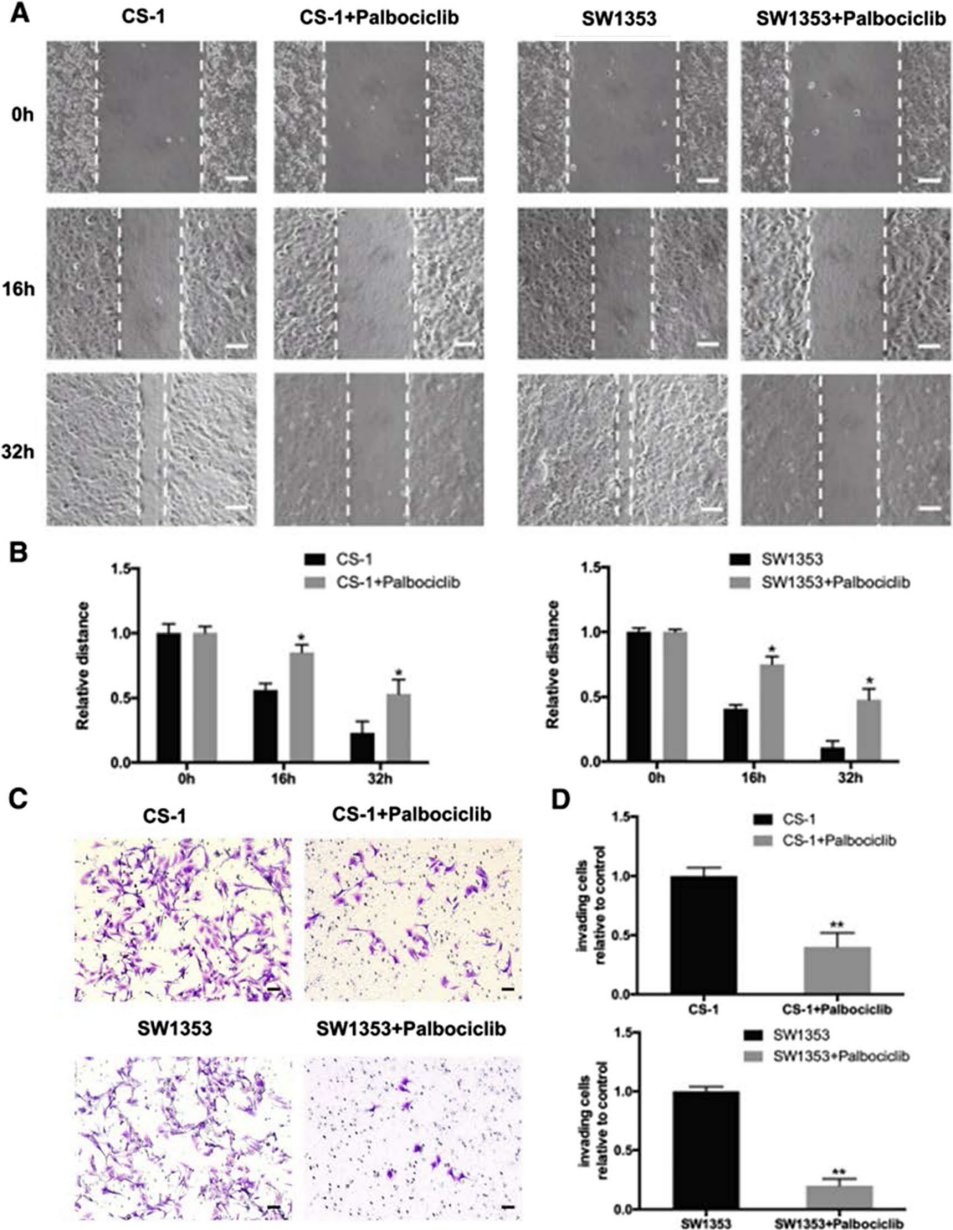
CDK4 inhibition induced by palbociclib suppresses cell migration and invasion. After exposure to 1 µM of palbociclib for the indicated time, the cell migration of CS-1 and SW1353 cells was determined by wound healing assay. **A** Representative images of CS-1 and SW1353 cell migration after palbociclib treatment for 0, 16, and 32 h (scale bar = 50 μm). **B** Cell migration distance of CS-1 cells was measured after palbociclib treatment. *P < 0.05 compared with 0 h group. **C** Human chondrosarcoma cells CS-1 and SW1353 were starved for 12 h, and then seeded in the top chambers of transwells with matrigel in the presence of the indicated doses of palbociclib. The bottom chambers of the transwells were filled with a medium containing 10% FBS. Cancer cells were allowed to invade for 10–12 h. The invading purple-stained cells showing irregular shape were photographed and counted (scale bar = 50 μm). **D** Quantitative analysis of the percentage of cell invasion using ImageJ. Columns represent the means of experiments performed in triplicate, where the bars represent the SD. **P < 0.01 compared with cell only group
